# Portal pressure is of significant prognostic value in primary biliary cholangitis

**DOI:** 10.1111/liv.15289

**Published:** 2022-06-01

**Authors:** Thomas W. Warnes, Stephen A. Roberts, Alexander Smith, Victor M. Cope, Patricia Vales, Raymond McMahon

**Affiliations:** ^1^ Liver Unit, Department of Gastroenterology Manchester Royal Infirmary Manchester UK; ^2^ Division of Population Health Health Services Research and Primary Care, University of Manchester Manchester UK; ^3^ Department of Radiology Manchester Royal Infirmary Manchester UK; ^4^ Department of Medical Physics Manchester Royal Infirmary Manchester UK; ^5^ Department of Histopathology Manchester Royal Infirmary Manchester UK

**Keywords:** portal pressure liver cirrhosis biliary

## Abstract

**Background and Aims:**

In other forms of chronic liver disease, measurement of portal pressure is of prognostic value, but this has not yet been established in primary biliary cholangitis (PBC). The aim of the study is to determine the prognostic value of hepatic venous pressure gradient (HVPG) in relation to liver‐related survival outcomes, as well as to the development of hepatic decompensation, oesophageal varices and variceal bleeding.

**Methods:**

Baseline HVPG and liver biopsies were obtained in 86 patients followed for 10 years in a controlled trial of colchicine treatment, and subsequently in a long‐term observational cohort study for a further 30 years.

**Results:**

There were 49 Hepatic deaths in addition to 10 Liver Transplants (Hepatic death/transplant; *n* = 59). Some of these were associated with a significant variceal bleed within 3 months of death or transplant (Portal hypertension‐associated death or transplant; *n* = 19). There were 63 deaths from all causes. During follow‐up, oesophageal varices developed in 26 patients, whilst 17 bled from varices and 32 developed hepatic decompensation over a median follow‐up of 18.1 years (1.9–28.5). Baseline HVPG was highly predictive of all 6 clinical outcomes and contributed significant predictive information additional to that provided by Mayo score and Ludwig stage.

**Conclusion:**

Measurement of baseline portal pressure is of significant prognostic value in primary biliary cholangitis.

**Abbreviations**
AMA: anti‐mitochondrial antibody
APRI: aspartate aminotransferase to platelet ratio index
CSPH: clinically significant portal hypertension
GOV: gastro‐oesophageal varices
HCC: hepatocellular carcinoma
HVPG: hepatic venous pressure gradient
MELD: Model for End‐Stage Liver Disease
PBC: primary biliary cholangitis
PHG: porto‐hepatic gradient
UDCA: ursodeoxycholic acid

**Key Points/Lay Summary**

The lack of hard therapeutic end points has led to controversy concerning the effectiveness of drug treatments for primary biliary cholangitis. Portal hypertension is associated with all the major complications of this disease and the measurement of portal pressure is shown in this study to be of significant prognostic value in relation to these complications and also to transplant‐free survival. It may therefore provide a valuable therapeutic end point in trials of new drug treatments for this condition.

## 1 INTRODUCTION

Around 40 per cent of patients with PBC, particularly those with elevated serum bilirubin levels and stage 3 or 4 disease histologically, fail to respond to ursodeoxycholic acid (UDCA)^1^ and are at risk of progression to cholestatic liver failure associated with complications of portal hypertension including bleeding oesophageal varices, encephalopathy, hepato‐renal syndrome, hepatocellular carcinoma and ultimately, cholestatic liver failure with progressively rising serum bilirubin levels. For such patients, second‐line treatments such as obeticholic acid^2^and fibrates^3^ need to be considered and prognostic models developed and validated in patients treated with UDCA, such as Paris 1^4^ and the UK—PBC risk score^5^ and Globe score,^6^ are not applicable.

Recent studies have clarified the histological correlates and prevalence of portal hypertension in PBC. This commences in the early stages of the disease, long preceding both rises in serum bilirubin and the development of cirrhosis and around 34% of patients with pre‐cirrhotic PBC have “high‐risk” portal hypertension, defined as an HVPG greater than 12 mmHg.^7^ Portal hypertension is related to cholestasis, interface hepatitis and portal tract and sinusoidal fibrosis and Ludwig stage shows a significant correlation with clinical outcomes such as transplant‐free survival and hepatic decompensation.^8^

Whilst there have been a number of studies documenting the prevalence and development of oesophageal varices and variceal bleeding in PBC, no measurements of portal pressure were reported in these studies.^9^, ^10^, ^11^ In other forms of chronic liver disease, evidence supporting a correlation between portal pressure, the presence and size of varices and the subsequent occurrence of variceal bleeding is conflicting.^12^, ^13^

Measurement of the HVPG is a safe and reproducible technique to assess portal pressure, and is the method of choice in PBC.^7^;^14^ In other forms of chronic liver disease, it is recognised to be of major value in assessment of prognosis, monitoring efficacy of medical treatment and evaluation of progression of portal hypertension^15^, ^16^, ^17^ but such data for PBC is largely lacking.

The present study aims to evaluate the prognostic value of portal hypertension in a large cohort of PBC patients with long‐term follow‐up.

## 2 PATIENTS AND METHODS

### 2.1 Patients

The patients in the present study were drawn from 104 patients who entered into the first trial of colchicine in August 1979,^18^ with a long‐term follow‐up of up to 40 years. Final data collection occurred in September 2019; the recently reported clinical outcomes included 73 liver‐related events.^8^ The first 10 years of the study comprised a controlled trial during which patients were randomly allocated to colchicine or placebo; no patients received UDCA prior to, or during the controlled phase of the study, which was performed in accordance with the ethical principles of the Declaration of Helsinki and was approved by the Hospital Ethics Committee of the Manchester Royal Infirmary. Written informed consent was obtained from each patient. Since no significant differences emerged in the outcomes between the two groups, these were combined for the purpose of the present report. The diagnosis of PBC was based on the finding of a raised serum alkaline phosphatase (ALP) of hepatic origin,^19^ a positive AMA test and a liver biopsy compatible with, or diagnostic of, PBC and an ultrasound scan showing no evidence of alternative pathology. Supportive drug therapy included supplementary vitamin D, monitored as necessary by measurement of 25 OH vitamin D levels,^20^ and cholestyramine for pruritus. Patients were followed in a Liver outpatients clinic and follow‐up data were collected every 3–6 months at which time clinical assessment of jaundice, oedema, ascites and hepatic encephalopathy were made and fasting blood was collected for haematological and biochemical parameters. Serum immunoglobulins as well as antimitochondrial antibody, antinuclear factor and smooth muscle antibodies were assessed at study entry, then yearly.

Following the termination of the trial, patients were offered colchicine or UDCA as part of their continuing care, since meta‐analyses had demonstrated no clear‐cut evidence of therapeutic benefit of any treatment option available at that time.^21^, ^22^ Further follow‐up data were extracted from patient notes until September 2019.

Patients were referred for consideration of liver transplantation if they were younger than 65 years of age with a Child‐Pugh score of greater than 8 or serum bilirubin greater than 100 micro mols per litre, thus according to AASLD Practise Guidelines. Since Manchester is not a designated UK transplant centre, such patients were referred to one of two National Liver Transplant Centres, where the final decision and timing regarding transplantation were made.

All patients who had a baseline assessment of HVPG were included in this study.

### 2.2 Outcomes

The cause of death was confirmed from hospital records, supported by death certificates and in many cases by autopsy findings, and outcomes were classified as follows:

1. Hepatic death/liver transplant (*n* = 59). The 49 deaths from cholestatic liver failure included 3 patients with combined P.B.C./Hepatocellular carcinoma. In addition, there were 10 liver transplants.
2. Portal hypertensive death/ transplant: defined by the presence of bleeding varices within 3 months of death or liver transplant (*n* = 19)
3. All deaths (overall survival). The median (IQR) age at death was 69 (63–74).
  Additional outcomes related to disease progression evaluated were:
4. The first episode following trial entry of hepatic decompensation, defined as clinical jaundice or a serum bilirubin of greater than 49 μmoL/L, encephalopathy (defined by confusion and flapping tremor supplemented by a numbers connection test, handwriting chart and if necessary, a blood ammonia),^23^ ascites confirmed on ultrasound, or clinically significant oedema.
5. The development of gastro‐oesophageal varices.
6. Variceal bleeds confirmed at endoscopy.

Outcomes were defined as the time to the first event in those in whom the event had not occurred before the baseline assessment.

### 2.3 Measurement of HVPG

This was performed as previously described.^7^ In brief, all drugs known to affect portal pressure were discontinued for 3 days prior to the study. The normal range for our laboratory is 1–5 (mean 2.3 mmHg) established in 17 patients without liver disease undergoing right‐sided cardiac catheterization and using a 6 or 7F standard balloon occlusion catheter. Hepatic vein catheterization was undertaken via the right femoral vein using the Seldinger technique. A 6 or 7F catheter was guided under fluoroscopic control into the main right hepatic vein and 3 measurements of both wedged (WHVP) and free (FHVP) hepatic venous pressure were taken. Wedged pressure was checked both before and after injection of contrast medium to confirm wedging of the catheter. Zero reference points were taken as the pressure in a free position in a large hepatic vein, and also on withdrawal in the inferior vena cava. Portal hypertension was diagnosed when the mean gradient between WHVP and FHVP (the hepatic venous pressure gradient, HVPG) was greater than 5 mmHg.

### 2.4 Liver biopsy

Pre‐entry percutaneous liver biopsies were performed at, or within 6 months before, study entry after informed consent and using a Trucut needle. The Ludwig system, which is of more prognostic value than the Nakanuma staging system,^8^ was used to assess disease stage. Liver biopsy and HVPG measurement were normally performed during the same 3‐day period of hospitalisation.

### 2.5 Clinical assessment

Routine clinical assessment at baseline included standard biochemical, haematological and immunological parameters and the computation of the Child‐Pugh class and Mayo score.^8^, ^24^

### 2.6 Statistical analysis

Patients were censored at death or last follow‐up assessment. Median follow‐up times were computed in those who had not had the event in question. Kaplan–Meier survival curves were used to visualise the time to event for the 6 outcomes, with the HVPG trichotomised as normal (≤5 mmHg), raised (>5 and ≤ 12 mmHg) or high‐risk (>12 mmHg) as previously.^7^

Formal analysis of prognostic potential utilised Cox regression models. Univariate models estimated the hazard ratio HR per mmHg HVPG along with its associated 95% confidence interval (CI) and P‐value. Further models estimated the effect of HVPG in models that adjusted (a) for Mayo score (as a continuous variable) and (b) both Mayo score and Ludwig stage (categorical). Patients who had encountered the event prior to baseline were excluded from the analyses of that event.

All analyses were conducted in the R statistical environment (version 4.0).

## 3 RESULTS

### 3.1 Patient characteristics

Eighty‐six of the 104 patients recruited to the trial had an HVPG assessment and were included in this study. Table 1 shows the characteristics of these patients, which are representative of the wide spectrum of clinical presentations and disease severity seen in a P.B.C. referral clinic. Overall,25% were cirrhotic (Ludwig Stage 4) and 6% were Childs‐Pugh class C. The median duration of time from the first diagnosis of P.B.C. to entry to the study was 12 months (range 1–119), and these characteristics did not differ substantively from those displayed by the 18 patients who did not consent to HVPG assessment. The number of events and median follow‐up time for each outcome are shown in Table 2; the median follow‐up was 13.1 years (IQR 1.7–28.5). Mayo score and Ludwig stage show their expected prognostic value in this dataset (Tables S1a,b). The rate of transplantation relative to Hepatic death in the present study is rather lower than might be expected; this is probably a reflection of the mean age of 59 at recruitment, together with the higher barriers to transplantation seen in the 1980s and 1990s. The median age at death was 69(63–74), whilst the mean was 68.

**TABLE 1:** Characteristics of patients and biopsies at the time of baseline HVPG measurement

Patients	86
Biopsies	85
Age	59.2 (52.2–64.9) [86]
Male	6/86 (7%)
Classification	
Presymptomatic	14/86 (16%)
Symptomatic	56/86 (65%)
PBC/AIH overlap	5/86 (6%)
Portal hypertensive PBC	5/86 (6%)
Atypical PBC	6/86 (7%)
Childs class	
A	55/83 (66%)
B	23/83 (28%)
C	5/83 (6%)
Mayo Score	5.6 (4.7–6.7) [80]
Ludwig Stage	
1	14/85 (16%)
2	15/85 (18%)
3	35/85 (41%)
4	21/85 (25%)
Albumin (g/l)	36.0 (33.0–40.0) [86]
Globulin (g/l)	40.0 (37.0–46.0) [86]
Bilirubin (μmol/l)	19.5 (11.2–38.8) [86]
c ALP (% ULN)	323 (168–458) [85]
ALT (g/l)	67 (42–95) [83]
AST (g/l)	77 (43–116) [59]
AMA titre	300 (300–1000) [80]
IgG (g/l)	17.1 (13.7–22.7) [84]
IgM (g/l)	5.0 (2.9–7.4) [84]
Hb (g/dl)	12.4 (11.3–13.6) [85]
WBC (x10^9^/l)	5.3 (4.0–7.2) [85]
Platelets (x10^9^/l)	231 (137–280) [84]
Prothrombin Index	1.0 (1.0–1.1) [86]

*Note*: Data represent median (IQR) [N] or number (%).

Abbreviations: cALP (% ULN), Serum Alkaline phosphatase as % upper limit of normal; AMA titre, reciprocal of antimitochondrial antibody titre; PBC, Primary Biliary Cholangitis; PBC/AIH overlap, Primary Biliary Cholangitis/autoimmune hepatitis overlap syndrome; Portal Hypertensive PBC, presentation with bleeding oesophageal varices in the absence of jaundice; Atypical PBC, AMA negative PBC or PBC with an initially normal serum alkaline phosphatase.

**TABLE 2:** Numbers of events and patients at risk for each outcome along with the median (inter‐quartile range) length of followup

	Pre‐baseline events	Never assessed	At risk	Post‐baseline events	Followup years Median (IQR)
All deaths	0	0	86	63	13.1 (1.7–28.5)
Hepatic death/transplant	0	0	86	59 (49 deaths, 10 Transplants)	13.1 (1.7–28.5)
PHT‐associated death/transplant	0	0	86	19 (18 deaths, 1 Transplant)	13.1 (1.7–28.5)
Decompensation	25	0	61	32	18.1 (1.9–28.5)
Varices	19	6	61	26	15.3 (1.6–28.5)
Variceal bleeds	8	3	75	17	16.4 (1.6–28.5)

*Note*: PHT associated death—liver‐related death associated with bleeding varices within 3 months of death. P.B.C. associated death—Death from cholestatic liver failure or liver‐related death associated with bleeding varices within 3 months of death. Excludes liver transplants but includes 3 HCC's and 6 co‐morbidities (see text).

### 3.2 Survival

**FIGURE 1:**
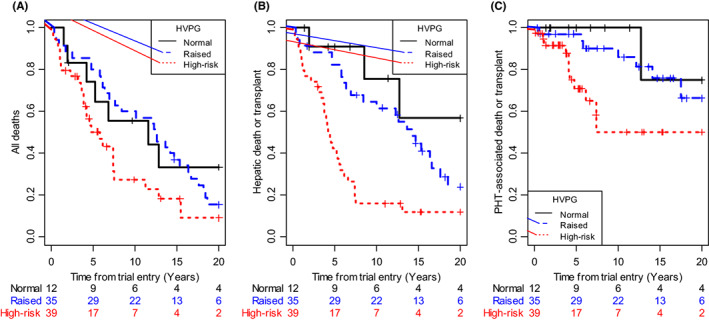
Patient survival. Kaplan–Meier plots of time to (A) any‐cause death, (B) Hepatic death or liver transplant, (C) PHT‐associated death by HVPG expressed as normal (≤5 mmHg), raised (>5 and ≤ 12 mmHg) or high‐risk (>12 mmHg). Numbers at risk at selected time points are shown below the plots. Hazard ratios and significance levels from the formal analysis are given in Table 3.

Figure 1 shows survival curves for the two specific causes of death and for all‐cause death. Table 3 gives the estimates of the effect of HVPG on survival based on the Cox models without and with adjustment for the conventional prognostic indicators, Mayo score and Ludwig stage. HVPG is strongly predictive of hepatic death /transplants and retains significant predictive power beyond that of the conventional predictors (HR = 1.08 [1.02–1.15] per mmHg, *P* = 0.011). Similarly, it is predictive of PHT associated death/ transplant (HR = 1.13 [1.02–1.25]), although this just fails to reach the conventional level of significance after allowing for Mayo score and Ludwig stage adjustments (*P* = 0.058). HVPG is predictive of all‐cause death but adds little to the conventional factors.

**TABLE 3:** HVPG as a prognostic factor

Outcome	Unadjusted		Adjusted for Mayo		Adjusted for Mayo and Ludwig	
	HR	*P*	HR	*P*	HR	*P*
All deaths	1.08 (1.03 to 1.14)	0.003	1.02 (0.96 to 1.08)	0.48	1.03 (0.96 to 1.10)	0.40
Hepatic death/transplant	1.11 (1.06 to 1.17)	<0.001	1.07 (1.02 to 1.14)	0.012	1.08 (1.02 to 1.15)	0.011
PHT death/transplant	1.13 (1.02 to 1.25)	0.015	1.12 (0.98 to 1.28)	0.072	1.12 (0.99 to 1.28)	0.058
Decompensation	1.18 (1.10 to 1.28)	<0.001	1.16 (1.06 to 1.27)	<0.001	1.21 (1.07 to 1.36)	0.001
Varices	1.12 (1.06 to 1.19)	<0.001	1.08 (1.02 to 1.14)	0.014	1.08 (1.01 to 1.15)	0.026
Variceal bleed	1.12 (1.02 to 1.23)	0.022	1.07 (0.96 to 1.19)	0.20	1.08 (0.96 to 1.22)	0.19

*Note*: Cox regression analyses with and without adjustment for Mayo score (continuous variable) and Ludwig stage (categorical). Hazard ratios (HR) and associated 95%CI per mmHg HVPG and *P*‐values. PHT associated death—liver‐related death associated with bleeding varices within 3 months of death. P.B.C. associated death—Death from cholestatic liver failure or liver‐related death associated with bleeding varices within 3 months of death. Excludes liver transplants but includes 3 HCC's and 6 co‐morbidities (see text).

Visually (Figure 1), it appears that most deaths are associated with the highest levels of HVPG, the high‐risk group. However, there is insufficient data to draw firm conclusions as to the shape of the relationship between pressure and survival.

### 3.3 Progression

**FIGURE 2:**
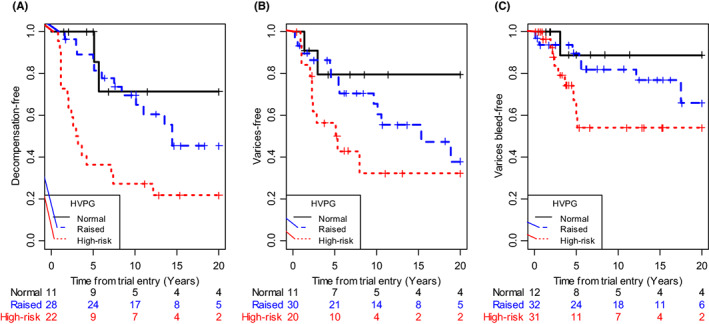
Disease progression. Kaplan–Meier plots of time to (A) decompensation, (B) varices, (C) bleeding from varices by HVPG expressed as normal (≤5 mmHg), raised (>5 and ≤ 12 mmHg) or high‐risk (>12 mmHg). Numbers at risk at selected time points are shown below the plots. Hazard ratios and significance levels from the formal analysis are given in Table 3.

Figure 2 shows the survival curves for the 3 markers of disease progression, with Table 3 giving the estimated prognostic effect sizes. HVPG is a strong predictor of decompensation, even after allowing for Mayo score and Ludwig stage (HR = 1.21 [1.07–1.36] per mmHg). Varices and bleeding from varices show a similar risk (HR = 1.08 (1.01–1.15 and 1.08 [0.96–1.22] per mmHg respectively), although with the smaller numbers we cannot demonstrate statistical significance for bleeds (*P* = 0.19).

Visually (Figure 2) the effect size seems more linear than for survival, but again there is insufficient data to draw firm conclusions as to the shape of the relationship between pressure and progression.

## 4 DISCUSSION

Portal hypertension is a frequent and serious complication of PBC, being present at baseline in 86% of patients in the present series. It starts early in the course of the disease, long preceding both rises in serum bilirubin levels and the development of cirrhosis.^7^ Clinically significant(“high‐risk”) portal hypertension has been defined as an HVPG>12 mmHg and in the current study was present at baseline in 34% of pre‐cirrhotic patients.

Both liver biopsy and measurement of portal pressure are invasive investigations which are not currently part of the routine management of PBC. However, whilst liver biopsy has a small but significant morbidity rate with occasional reports of mortality, measurement of HVPG is associated with neither complication. Therapeutic trials in PBC typically recruit a homogenous set of patients with early disease who have normal or minimally elevated serum bilirubin levels and have never exhibited features of decompensation or bled from varices. The participants in the present study, in contrast, reflect that PBC is, in reality, a heterogenous disease, with atypical features present in between 38% and 65% of cases^25^ whilst the inclusion of patients with all degrees of disease severity not only represents a more representative sample but has afforded greater power to detect associations given the relatively small sample size.

Whilst measurement of HVPG can provide prognostic information additional to that available from non‐invasive models in a wide variety of other chronic liver diseases complicated by the development of portal hypertension,^15^, ^16^, ^17^ it is not recommended under current guidelines in the management of PBC.^1^ This is because it has not previously been shown to be of prognostic value in this disease, and because numerous non‐invasive prognostic models already exist largely based on biochemical response criteria for UDCA treated patients. However, for patients who fail to respond, and ultimately decompensate, alternative therapeutic end points are required and the best model currently available is Mayo score which is independent of the treatment used.^8^, ^26^ It is in this group that HVPG measurement may be of particular value.

### 4.1 Survival

HVPG measurement is of significant prognostic value in predicting survival in many forms of cirrhosis. In a survey of 10 studies of cirrhosis of mainly alcoholic origin the prediction of survival based on conventional criteria was significantly improved by information obtained from hepatic vein catheterisation^27^ and an HVPG level of 15 mmHg has been identified as an independent prognostic determinant of survival.^28^ In the only comparable study in PBC, pre‐entry portal pressure measured by the porto‐hepatic gradient (PHG) was significantly correlated with long‐term survival^29^; however, the initial Mayo score remained the best predictor. The present study shows that not only do PBC patients with increased portal pressure have a significantly reduced transplant‐free survival, as well as increased risks of portal hypertensive death, but that these conclusions still hold after allowance for both Ludwig stage and Mayo score. The different conclusions reached by the two PBC studies may be related to a difference in clinical material; the Huet study^29^ was confined to patients with earlier disease and those with a previous history of ascites, bleeding varices or porto‐systemic encephalopathy were excluded, resulting in a reduction in the number of outcomes studied.

### 4.2 Decompensation, varices and variceal bleeding

In other diseases associated with compensated cirrhosis an HVPG>10 mmHg represents clinically significant portal hypertension (CSPH)^30^ and is an independent predictor for hepatic decompensation, as well as for the development of varices and hepatoma, independent of MELD and Child‐Pugh scores.^31^ The level of portal pressure correlates with the degree of liver failure^32^ whilst the development of ascites probably requires a gradient above 8 mmHg.^27^ However, such data is lacking in PBC.

Decompensation in the present study is defined by the presence of one or more clinical features including jaundice, ascites, oedema or encephalopathy. Prior to entry 25 of the 86 patients had shown some features of decompensation, and the prognostic value of the baseline H.V.P.G. was therefore confined to the remaining 61 well‐compensated patients, 32 of whom decompensated during follow‐up. In this cohort, patients with elevated portal pressure were significantly more likely to decompensate than those with normal levels. Whilst bleeding from varices may legitimately be considered a feature of hepatic decompensation, it merits separate analysis because of the element of subjectivity in documenting the first onset of jaundice, oedema, ascites and encephalopathy which therefore represent relatively “soft” endpoints, in contrast to the easily established date of the first bleed from varices, which is a “hard” endpoint.

The formation of oesophageal varices requires a portal pressure above 10 mmHg, whilst an HVPG of ≤12 mmHg offers almost complete protection against variceal bleeding and represents the therapeutic target for protection against first or recurrent bleeding from varices.^33^ Despite the lack of a linear relationship between portal pressure and the risk of bleeding in retrospective studies, most have concluded that the height of portal pressure is an important predictive factor for bleeding.^33^ Significantly, the above data is largely lacking in patients with PBC. In the present study, varices were present in 19 of the 80 (24%) patients who had been examined by endoscopy prior to entry to the study and were found in a further 26 patients during a median follow‐up of 18.1 years. This experience compares with those reported from the Mayo clinic where in one study varices developed in 31% of PBC patients over a median follow‐up of 5.6 years and 40% of these bled within 4 years of development,^9^ whilst in a second study varices were present in 23% of patients entering a UDCA trial, and new varices developed in 25% and 58% at 2 and 4 years respectively.^10^ A number of non‐invasive models are available to predict the presence of varices usually incorporating varying cut off levels for platelet count, with additional features including serum albumin and alkaline phosphatase (Newcastle Risk Score),^34^ the Baveno—V1 criteria for varices screening (based on platelet count and Fibroscan reading),^35^ whilst in another multivariate analysis a platelet count <140 000 and a Mayo risk score of 4.5 or greater were independent predictors of varices and identified patients who need a screening endoscopy.^11^ Whilst our study shows for the first time that in PBC patients an increased HVPG correlates strongly with the presence of varices, this finding is hardly surprising and its cost effectiveness in assessing the need for screening endoscopies needs to be evaluated against the non‐invasive models already available for this purpose.

Primary prophylaxis is the prevention of bleeding from varices in patients that have never bled. In forms of cirrhosis other than PBC if the HVPG is less than 12 mmHg, then the risk of bleeding is close to zero. Similarly, treatment with beta‐blockers with or without nitrates, used in the prevention of rebleeding in patients with varices reduces the risk of bleeding in those whose HVPG falls to less than 12 mmHg to nearly zero.^33^ In the present study, the 8 patients who had bled from varices prior to entry have been excluded from the analysis, which is confined to the 26 patients with no bleeds pre‐entry who bled during follow‐up(Table 2), and our findings confirm that, in relation to primary prophylaxis, a raised baseline HVPG correlates strongly with the risk of subsequent bleeding from varices.

### 4.3 Strengths and limitations

The strengths of this study are the long and systematic follow‐up of well‐defined patients in a trial context.

There are a number of limitations of our study, which started in 1980, since which time there have been significant changes both in the management of portal hypertension, in particular the introduction of non‐specific beta‐blockers, and also in the management of PBC, notably the introduction of UDCA as first‐line treatment for the disease. During the first 10 years of the study no patient received this drug and any confounding effect of UDCA on portal pressure and overall prognosis was therefore avoided. Following this, patients were offered UDCA or colchicine in a non‐randomised fashion, which allowed for long‐term follow‐up, but at the expense of possible treatment bias. We feel this is unlikely to have affected our conclusions: firstly, the vast majority of the clinical outcomes were reached during the first 10 years of the study; secondly, the effect of both UDCA and colchicine on clinical outcomes is controversial, with successive Cochrane reviews documenting little or no effect of either drug on survival.^36^, ^37^ In addition, studies have shown that the effect of UDCA on portal hypertension is unlikely to be significant,^36^, ^37^ and we found no effect of colchicine on portal pressure in the present study. The sample is relatively small, which precludes the determination of cutoffs or the development of reliable predictive models.

Finally, although the inclusion of patients with decompensation at entry to the study is debateable, it has, as discussed, some definite advantages. Furthermore, we have conducted a sensitivity analysis which included only compensated patients and the estimates of the effects of HVPG are essentially unchanged (Table S2).

In summary, this study shows for the first time that baseline HVPG is of significant prognostic value in PBC in relation to all three survival outcomes, as well as to the risks of decompensation, the development of varices and of variceal bleeding. Furthermore, it contributes prognostic information additional to that provided by Mayo score in relation to hepatic death/liver transplant, as well as to hepatic decompensation, whilst baseline portal pressure retains this additional prognostic value when adjusted for both Mayo score and Ludwig stage. Its cost effectiveness now needs to be evaluated against appropriate non‐invasive models such as APRI score and Transient Elastography.^1^, ^38^

## CONFLICT OF INTEREST

The authors declare no conflict of interest and no source of funding.

## ETHICAL STATEMENT

This study was approved by the Medical Ethics Committee of the Manchester Royal Infirmary and conforms to the requirements of the Declaration of Helsinki. Written informed consent was obtained from the patients involved in this study.

## Supporting information


Tables S1‐S2

## Data Availability

The data that support the findings of this study are available on request from the corresponding author. The data are not publicly available because of privacy or ethical restrictions.
