# Supplementary material for: Portal pressure is of significant prognostic value in primary biliary cholangitis
Source: Liver Int. 2022 Jun 1;43(1):139–46. doi: 10.1111/liv.15289 (PMC10084443; doi:10.1111/liv.15289)
Supplement: Supplementary file 1 — Tables S1‐S2 [file LIV-43-139-s001.docx]

**Supplementary tables S1a and S1b: Ludwig and Mayo as prognostic indicators**

Univariate Cox models stratified by Child score

| **1a) Mayo score:** Hazard ratios per point | | |  |  |
| --- | --- | --- | --- | --- |
| **Outcome** | **Mayo** | **HR** | **P** | **missing** |
| All deaths | per point | 3.45 (2.25 to 5.31) | <0.001 | 6 |
| Hepatic death/transplant | per point | 3.21 (2.06 to 4.99) | <0.001 | 6 |
| PHT-associated death | per point | 3.37 (1.42 to 7.99) | 0.002 | 6 |
| Decompensation | per point | 2.42 (1.38 to 4.23) | 0.001 | 6 |
| Varices | per point | 2.88 (1.89 to 4.39) | <0.001 | 6 |
| Variceal bleed | per point | 2.54 (1.21 to 5.34) | 0.009 | 5 |

| **1b) Ludwig Stage:** Hazard ratios relative to stage 2 | | |  |  |
| --- | --- | --- | --- | --- |
| **Outcome** | **Stage** | **HR** | **P** | **missing** |
| All deaths | 1 | 0.49 (0.16 to 1.48) | 0.001 | 1 |
|  | 2 | 1 (reference) |  |  |
|  | 3 | 2.21 (1.05 to 4.64) |  |  |
|  | 4 | 2.59 (1.13 to 5.92) |  |  |
| Hepatic death/transplant | 1 | 1.87 (0.5 to 7.09) | <0.001 | 1 |
|  | 2 | 1 (reference) |  |  |
|  | 3 | 4.98 (1.7 to 14.6) |  |  |
|  | 4 | 7.38 (2.39 to 22.76) |  |  |
| PHT-associated death | 1 | 0 | 0.002 | 1 |
|  | 2 | 1 (reference) |  |  |
|  | 3 | 8.17 (1.05 to 63.71) |  |  |
|  | 4 | 6.69 (0.71 to 62.65) |  |  |
| Decompensation | 1 | 2.7 (0.48 to 15.05) | 0.005 | 1 |
|  | 2 | 1 (reference) |  |  |
|  | 3 | 5.46 (1.23 to 24.21) |  |  |
|  | 4 | 9.04 (1.93 to 42.35) |  |  |
| Varices | 1 | 0.34 (0.03 to 3.44) | <0.001 | 1 |
|  | 2 | 1 (reference) |  |  |
|  | 3 | 7.52 (2.03 to 27.87) |  |  |
|  | 4 | 12.9 (3.25 to 51.23) |  |  |
| Variceal bleed | 1 | 0 | <0.001 | 1 |
|  | 2 | 1 (reference) |  |  |
|  | 3 | 11.33 (1.43 to 89.9) |  |  |
|  | 4 | 8.06 (0.86 to 75.18) |  |  |

^*^ Note that we use stage 2 as a reference level as some outcomes have no events for stage 1 patients.

**Supplementary Table S2**: HVPG as a prognostic factor. Sensitivity analysis excluding those patients who were decompensated at baseline.

|  | **Unadjusted** | | **Adjusted for Mayo** | | **Adjusted for Mayo and Ludwig** | |
| --- | --- | --- | --- | --- | --- | --- |
| **Outcome** | **HR** | **P** | **HR** | **P** | **HR** | **P** |
| All deaths | 1.13 (1.05 to 1.21) | <0.001 | 1.03 (0.94 to 1.12) | 0.54 | 1.05 (0.96 to 1.15) | 0.31 |
| Hepatic death/transplant | 1.19 (1.10 to 1.29) | <0.001 | 1.14 (1.04 to 1.25) | 0.004 | 1.17 (1.06 to 1.30) | 0.002 |
| PHT death/transplant | 1.17 (1.00 to 1.38) | 0.039 | 1.09 (0.90 to 1.31) | 0.38 | 1.22 (0.95 to 1.57) | 0.11 |
